# Supplementary material for: Single and binary protein electroultrafiltration using poly(vinyl-alcohol)-carbon nanotube (PVA-CNT) composite membranes
Source: PLoS One. 2020 Apr 16;15(4):e0228973. doi: 10.1371/journal.pone.0228973 (PMC7162463; doi:10.1371/journal.pone.0228973)
Supplement: S1 File — (PDF) [file pone.0228973.s001.pdf]

Single and binary protein electroultrafiltration using poly(vinyl-alcohol)-carbon nanotube (PVA-CNT) composite membranes

Protein electroultrafiltration using PVA-CNT composite membranes

Raymond Yeung[1], Xiaobo Zhu[2], Terence Gee[1], Ben Gheen[1], David Jassby[2], Victor G. J. Rodgers[1]*

[1]Department of Bioengineering, University of California, Riverside, Riverside, California, United States of America

[2]Department of Civil and Environmental Engineering, University of California, Los Angeles, Los Angeles, California, United States of America

* Victor G. J. Rodgers

E-mail: victor.rodgers@ucr.edu

Contents

A.1. PVA-CNT membrane images 3

A.2. Pore size analysis 6

A.3. Experimental system 7

A.4. Experimental procedures 8

A.4.1 Analysis of feed and sample 8

A.4.2 Global transmembrane zeta potential 8

A.5. Protein electrostatic potential 9

A.6. Flux and sieving for binary protein electroultrafiltration 10

A.7. Membrane hydraulic permeability 11

A.8. Fouling of PS-35 ultrafiltration membranes 12

A.9. Debye length calculation 13

A.10. Protein denaturation during electroultrafiltration 15

References 16

A.1. PVA-CNT membrane images

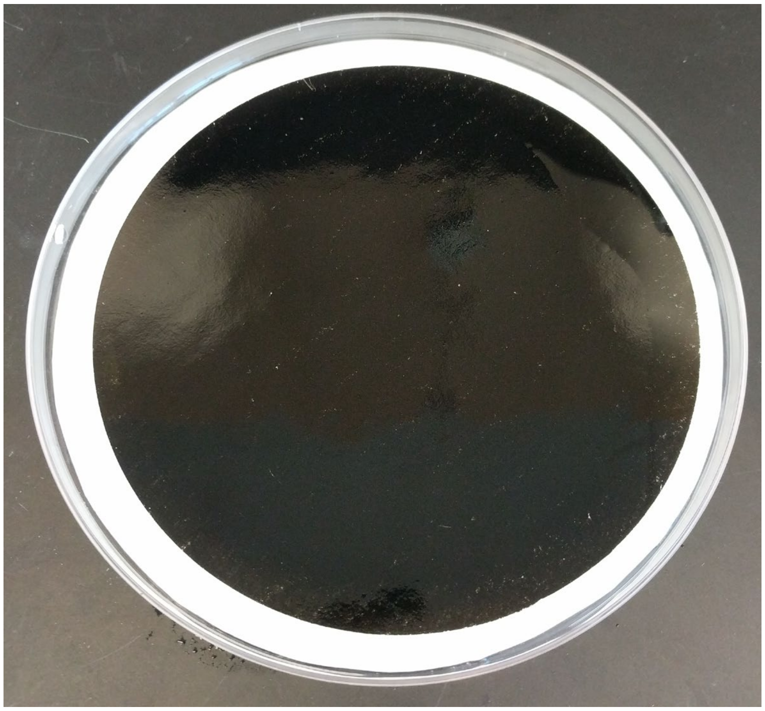

S1 Fig. Digital camera image of PVA-CNT PS-35 composite membrane.

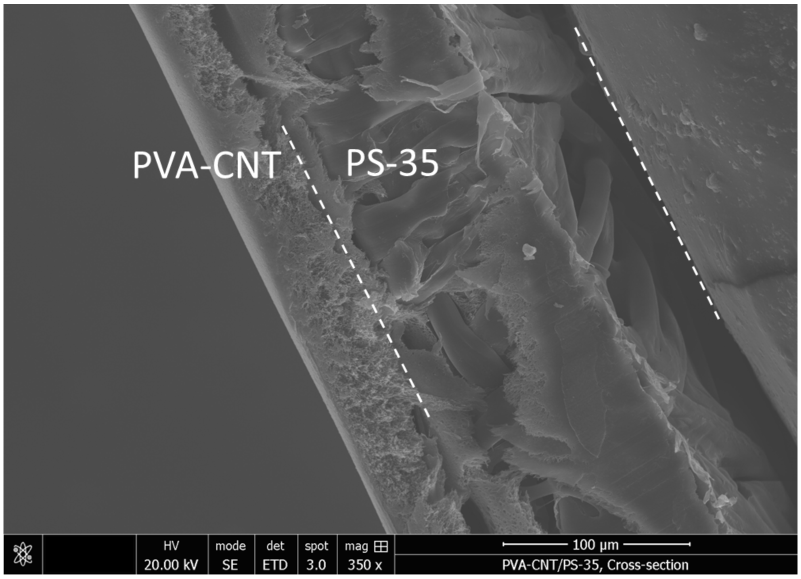

S2 Fig. SEM image of cross section of CNT layer deposited on a polysulfone UF membrane.

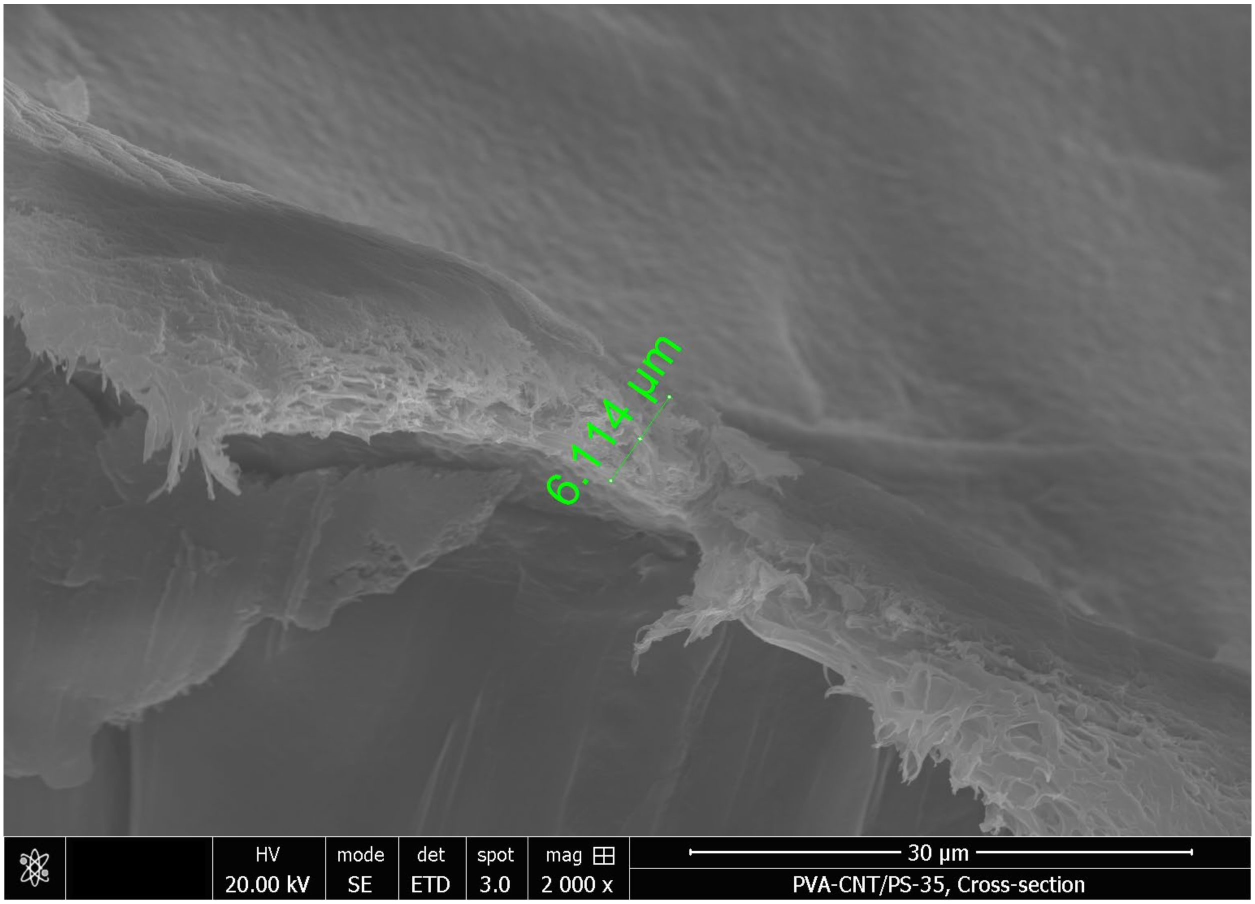

S3 Fig. Evaluation of PVA-CNT thin film thickness from SEM image of cross section of CNT layer.

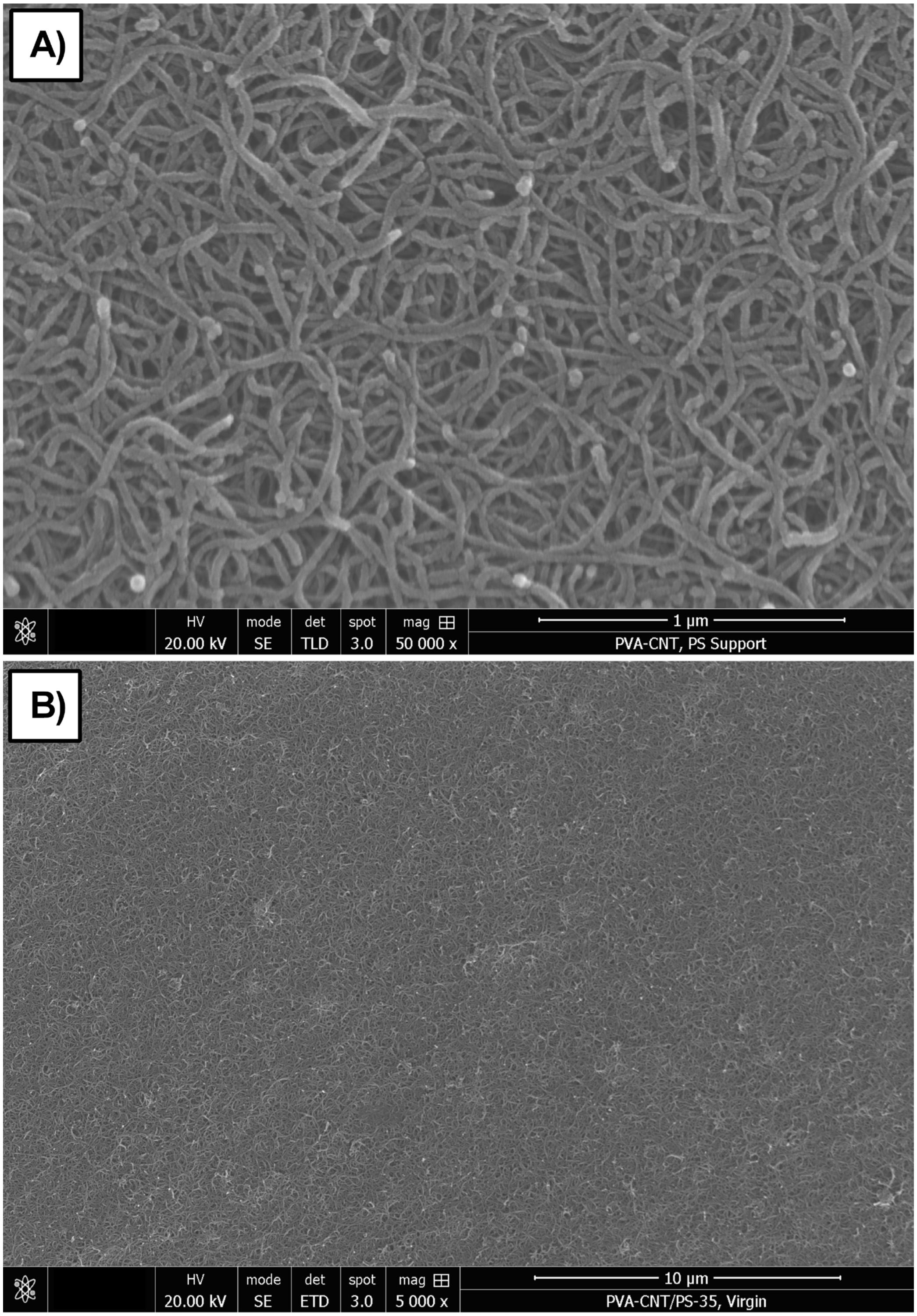

S4 Fig. SEM image of top view of a virgin PVA-CNT membrane at A) 50K magnification and B) 5K magnification.

A.2. Pore size analysis

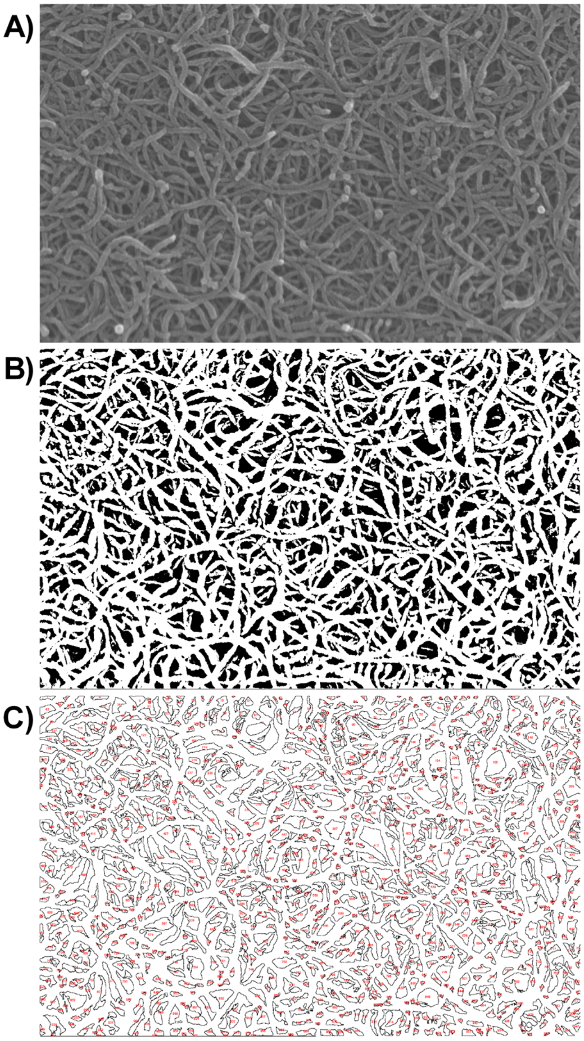

S5 Fig. Pore size analysis of PVA-CNT using ImageJ. A) Original PVA-CNT membrane surface image from SEM; B) Binary image from thresholding; C) Output of analyzed regions used for calculation of pore size.

Image analysis using ImageJ was used to determine the average pore size of the PVA-CNT layer. For detection of the regions used for the calculation, the minimum size was set to 0.007 μm^2 (to remove small features) and the circular parameter was set to 0.01 – 1. The average pore area was determined to be 0.177 μm^2. Assuming round pores, the typical pore diameter was determined to be approximately 475 nm.

A.3. Experimental system

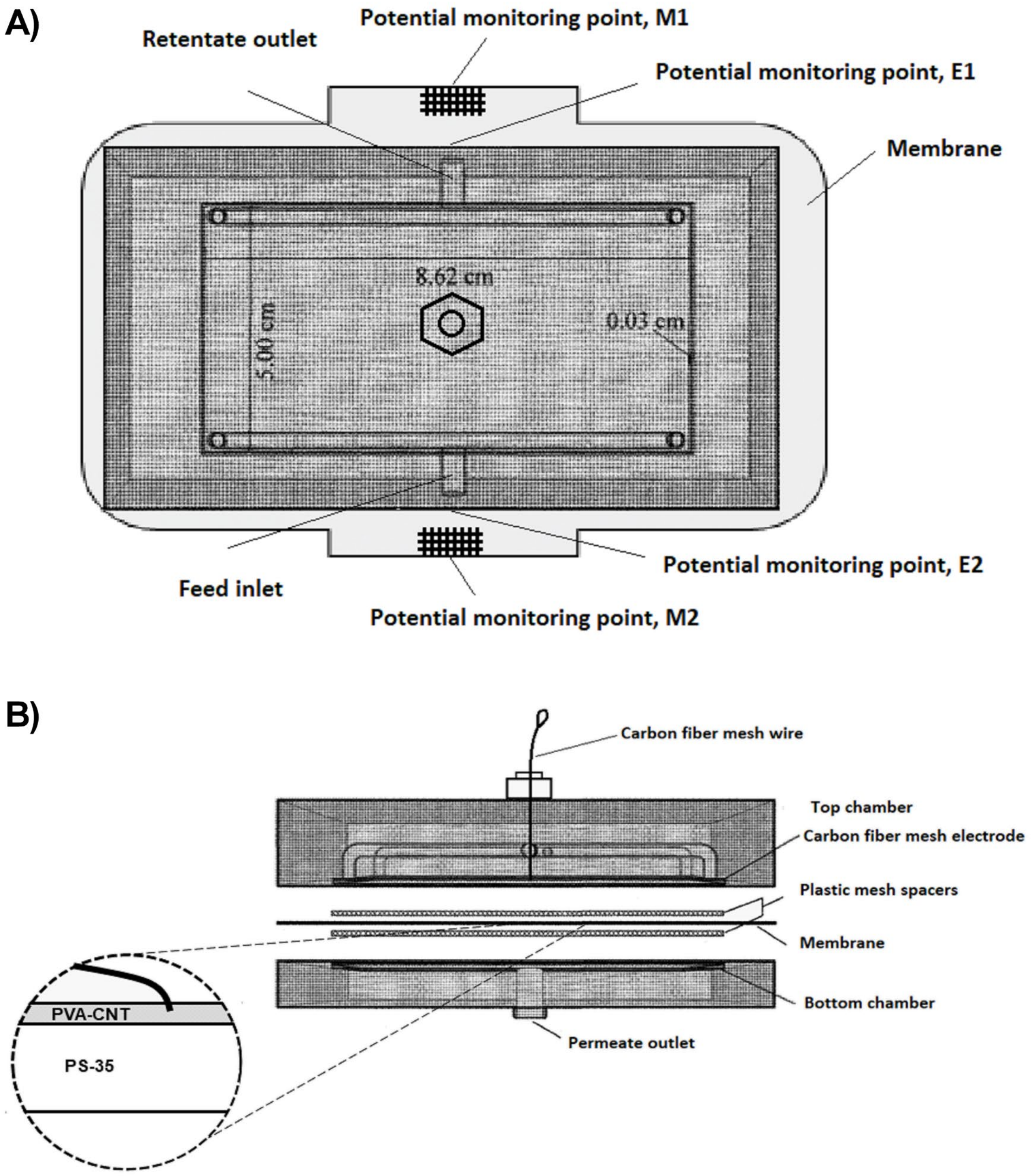

S6 Fig. Schematic of experimental flow cell. A) Top view of membrane in flow cell. The potential was monitored using a multimeter at each side of the membrane at locations adjacent to the edge of the flow cell and the edge of the membrane. B) Side view of the membrane flow cell. A plastic mesh spacer was placed between the PVA-CNT membrane and the carbon mesh electrode.

A.4. Experimental procedures

A.4.1 Analysis of feed and sample

Photometric measurements were used to evaluate the protein concentrations (Cary 50 Bio UV-VIS, Agilent, Santa Clara, CA, USA). For samples with a single protein, the protein concentration was determined using a bicinchoninic acid assay (SKU 23225, Thermo Scientific; Waltham, MA, USA) which is used for determining the total protein concentration in a solution. For binary protein studies (αLA and HEL), the total protein concentration was determined first using a bicinchoninic acid assay. The lysozyme concentration was evaluated using a lysozyme activity assay (LY0100, Sigma-Aldrich, Corp., St. Louis, MO, USA). The concentration of αLA was then determined from the difference in the evaluated total protein concentration and lysozyme concentration.

A.4.2 Global transmembrane zeta potential

The effective transmembrane zeta potential, ξ_{eff}, of the membranes was calculated based on the relation to the streaming potential difference, $\Delta\Psi_p$, and the pressure gradient, ΔP, using the Helmholtz-Smoluchowski equation [1]:

$$\frac{\Delta\Psi_p}{\Delta P} = \frac{\varepsilon_0 \varepsilon_r \zeta_{eff}}{\mu \Lambda_0} \tag{S1}$$

where ε_0 is the permittivity of free space (8.85×10^{-12} J V^{-2} m^{-1}), ε_r is the relative dielectric constant of the solution (78.6), μ is the solution viscosity (8.91×10^{-4} Pa s^{-1} at 25 °C), and Λ_0 is the solution conductivity (118.45×10^{-3} S m^{-1}) [2].

A.5. Protein electrostatic potential

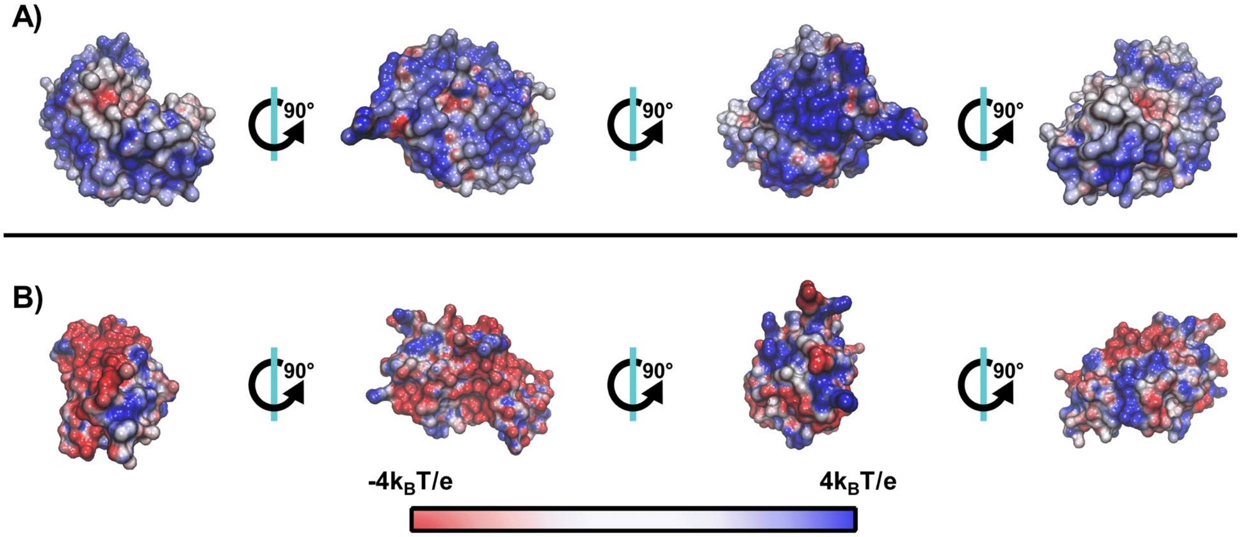

S7 Fig. Charge distribution of hen egg-white lysozyme and α-lactalbumin. Electrostatic potential visualization of A) hen egg-white lysozyme (PDB ID: 1HFZ) and B) α-lactalbumin (PDB ID: 1HFZ) from calculations using the APBS-PDB2PQR software suite [3] and generated using VMD [4]. The potentials are on a [-4 k_BT/e, 4 k_BT/E] red-white-blue color map. Calculations were performed at 4 mM ionic strength, 298.15 Kelvin, protein dielectric of 2, and solvent dielectric of 78. Although α-lactalbumin has a net negative charge, the protein contains positively charged regions which may bind to the negatively charged CNTs and the polysulfone membrane.

A.6. Flux and sieving for binary protein electroultrafiltration

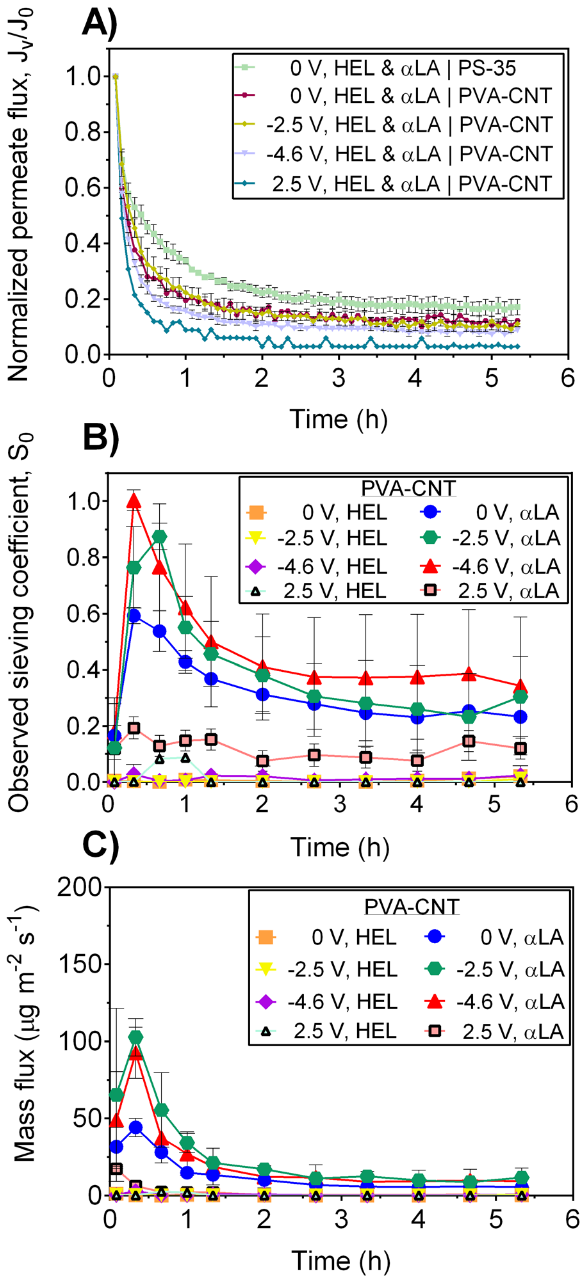

S8 Fig. Binary protein electroultrafiltration with application of anodic and cathodic potentials. A) Normalized permeate flux, B) observed sieving coefficient, and C) mass flux during EUF of binary protein solutions of 0.1 g/L αLA and 0.1 g/L HEL at TMP of 1 psi with different cathodic potentials (vs. Ag/AgCl) applied to the PS-35 (n=3) and PVA-CNT membranes (n=2; n=1 for 2.5 V). Error bars represent the standard error of the weighted mean.

A.7. Membrane hydraulic permeability

S1 Table. Hydraulic permeability for assessment of membrane fouling. Weighted average pre-experimental and post-experimental hydraulic permeability data of membranes used in EUF experiments (1 psi, 555 s-1 crossflow shear rate, 4 mM ionic strength, pH 7.4).

Protein feed	Applied potential vs. Ag/AgCl (V)	Membrane	Pre-experimental hydraulic permeability, $L_p \times 10^{10}$ ($m\ s^{-1}\ Pa^{-1}$)	Post-experimental hydraulic permeability, $L_p \times 10^{10}$ ($m\ s^{-1}\ Pa^{-1}$)	Percent change in hydraulic permeability (%)
0.1 g/L HEL	-	PS-35	12.2 ± 0.3	5.72 ± 0.08	-46 ± 3
	0	PVA-CNT	10.46 ± 0.07	2.16 ± 0.05	-75.7 ± 0.9
	-4.6	PVA-CNT	7.95 ± 0.09	1.76 ± 0.05	-80 ± 1
0.1 g/L αLA	-	PS-35	11.5 ± 0.3	4.82 ± 0.09	-31 ± 3
	0	PVA-CNT	11.6 ± 0.2	8.7 ± 0.2	-25 ± 2
	-4.6	PVA-CNT	10.95 ± 0.02	7.7 ± 0.2	-29 ± 2
0.1 g/L HEL & 0.1 g/L αLA	-	PS-35	10.4 ± 0.3	1.15 ± 0.02	-90 ± 3
	0	PVA-CNT	8.9 ± 0.4	0.36 ± 0.01	-95 ± 7
	-2.5	PVA-CNT	9.4 ± 0.4	0.59 ± 0.01	-95 ± 5
	-4.6	PVA-CNT	9.3 ± 0.1	1.01 ± 0.07	-89 ± 1

A.8. Fouling of PS-35 ultrafiltration membranes

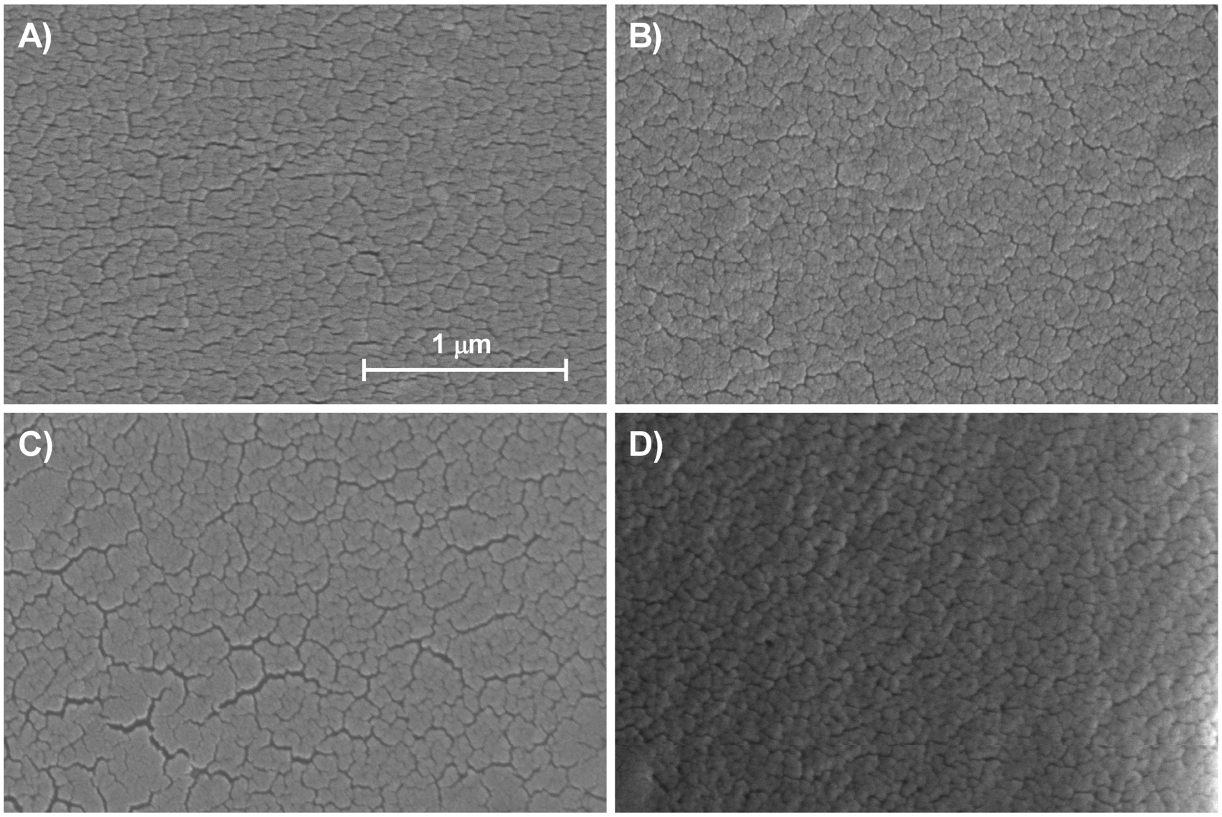

S9 Fig. SEM image of surfaces of uncoated PS-35 polymeric ultrafiltration membranes. A) virgin PS-35 membrane, B) PS-35 membrane following single protein HEL UF (0 V vs. Ag/AgCl), C) PS-35 membrane following single protein αLA UF (0 V vs. Ag/AgCl), D) binary protein UF of αLA and HEL(0 V vs. Ag/AgCl) with 0.1 g/L of each protein component; 555 s-1 crossflow shear rate; 1 psi TMP; 9.33 h duration.

A.9. Debye length calculation

For low surface potentials, say, $\Psi \leq 0.025$ V, the Debye length, κ^{-1}, is given by:

$$\kappa^{-1} = \left(\frac{e^2}{\varepsilon_r \varepsilon_0 k_B T} \sum_{i=1}^{N} z_i^2 n_{i\infty} \right)^{-1/2} \quad \text{(S2)}$$

where e is the elementary charge, ε_r is the dielectric constant, ε_0 is the permittivity of free space, k_B is the Boltzmann constant, T is the thermodynamic temperature, z_i is the valence of the ith ionic species, and $n_{i\infty}$ is the bulk ionic concentration [5].

The Debye length calculation for electric potential distributions for high surface potentials ($\Psi >$ 0.025 V) is given by:

$$\kappa^{*-1} = \left(\frac{e^2}{\varepsilon_r \varepsilon_0 k_B T} \sum_{i=1}^{N} z_{c,i}{}^2 n_{c,i\infty} \right)^{-1/2} \quad \text{(S3)}$$

where $z_{c,i}$ is the valence of the ith counterion species, and $n_{c,i\infty}$ is the bulk counterion concentration.

When charged particles interact, their diffuse electrical double layers overlap, resulting in repulsive electrostatic forces between them. The Debye length, κ^{-1}, is a measure of the electric double layer thickness, and is a property of the electrolyte solution. Although the actual thickness of the double layer extends well beyond κ^{-1}, the Debye length typically represents a characteristic distance from the charged surface to a point where the electric potential decays by about an order of magnitude from that of the surface potential, and provides a suitable parameter to characterize distances at which electrostatic interactions would be significant [6]. At the beginning of the EUF process, the surface potential of the cathodic PVA-CNT layer is high, thus a generalization can be made such that the counterions are the dominant ions in determining the surface charge behavior. As the electroultrafiltration proceeds, the PVA-CNT layer assumes the charge of the adsorbed protein. Equations S2 and S3 show the calculated Debye length for two cases: both ionic species

considered for low surface potential, and only the counterion species considered for the high surface potential. The calculated distances at which the electrostatic effects of the charged proteins (with low surface potential) and membrane (with high surface potential) persist are short relative to the large, typical pore size of the PVA-CNT network which is approximately 475 nm. Interestingly, for single protein EUF of α-LA at no applied electrical potential, single protein EUF of HEL at an applied cathodic potential of -4.6 V vs. Ag/AgCl, and binary protein EUF of HEL and αLA at an applied cathodic potential of -4.6 V vs. Ag/AgCl, the fouling layers observed from the SEM images of the upper surface of the electrically conducting membranes extended across the porous PVA-CNT network. The significant protein coverage of the upper PVA-CNT surface as observed by SEM, along with the transient flux and sieving results, suggests formation of multilayers of adsorbed protein which ultimately govern the protein transmission at extended stages of UF/EUF.

S2 Table. Debye length calculations at low and high surface potentials. Although the calculated Debye lengths are significantly smaller than the large pore sizes of the PVA-CNT network, the fouling observed on the surface extend across the porous PVA-CNT thin film which suggests formation of multilayers of adsorbed protein.

Surface potential, Ψ	Debye Length, κ^{-1} (nm)
Low $\Psi \leq 0.025$ V	4.58
High $\Psi > 0.025$ V	7.35

A.10. Protein denaturation during electroultrafiltration

Although the results of single and binary protein EUF using an electrically conductive CNT-polymer composite UF membrane show potential for modulating protein transmission for a short period, a natural concern for applications in protein filtration is whether the proteins are denatured during the process. Electrolysis, a concomitant to EUF, generates hydrogen gas at the cathode (reduction) and oxygen gas at the anode (oxidation). As a result, the local pH at the vicinity of the cathode is high which could contribute to denaturation of proteins. Saxena et al. [7] and Kappler et al. [8] investigated the possibility of denaturation in their independent studies of EUF of BSA and lysozyme using electrodes on opposite sides of the membrane with an applied electric field of 200 V m^{-1} (200 mM ionic strength) and 4000 V m^{-1} (5 mM ionic strength), respectively. Both groups did not observe denaturation for both proteins at extended lengths of filtration (3.5 h – 7 h). Moreover, Kappler et al. did not observe significant denaturation or reduction in enzyme activity of lysozyme when contacting any of the electrodes. Yang et al. [9] also did not detect a significant change in enzyme activity with pulsed electric fields from 0 to 38 kV cm^{-1} at moderate ionic strength (25 mM). While previous investigations suggest the applied electric field of 15 kV m^{-1} used in the current work results in no significant denaturation of lysozyme in the bulk phase, there may still exist denaturation or damage to the nanoscale layer close to the cathodic PVA-CNT porous network contributing to fouling of the membrane with denatured lysozyme proteins during EUF. Conversely, for EUF of α-lactalbumin, the elevated pH at the local regions of the PVA-CNT network with an applied electric potential increases the surface charge of α-lactalbumin resulting in reduced adsorption due to the greater electrostatic repulsive forces between the proteins.

References

1. Hunter R. Zeta Potential in Colloid ScienceAcademic Press. New York, USA. 1981.
2. Clogston JD, Patri AK. Zeta potential measurement. Characterization of nanoparticles intended for drug delivery: Springer; 2011. p. 63-70.
3. Dolinsky TJ, Nielsen JE, McCammon JA, Baker NA. PDB2PQR: an automated pipeline for the setup of Poisson–Boltzmann electrostatics calculations. Nucleic acids research. 2004;32(suppl_2):W665-W7.
4. Humphrey W, Dalke A, Schulten K. VMD: visual molecular dynamics. Journal of molecular graphics. 1996;14(1):33-8.
5. Hiemenz PC, Rajagopalan R. Principles of Colloid and Surface Chemistry, revised and expanded: CRC press; 1997.
6. Masliyah JH, Bhattacharjee S. Electrokinetic and colloid transport phenomena: John Wiley & Sons; 2006.
7. Saxena A, Tripathi BP, Shahi VK. An improved process for separation of proteins using modified chitosan–silica cross-linked charged ultrafilter membranes under coupled driving forces: Isoelectric separation of proteins. Journal of colloid and interface science. 2008;319(1):252-62.
8. Käppler T, Posten C. Fractionation of proteins with two-sided electro-ultrafiltration. Journal of biotechnology. 2007;128(4):895-907.
9. Yang R, Li S, Zhang Q. Effects of pulsed electric fields on the activity of enzymes in aqueous solution. Journal of Food Science. 2004;69(4):FCT241-FCT8.
